# Direct current cardioversion of atrial fibrillation in patients with left atrial appendage occlusion devices

**DOI:** 10.3389/fcvm.2025.1604268

**Published:** 2025-11-05

**Authors:** Xin Xie, Zijun Chen, Xiaorong Li, Jinbo Yu, Jian Zhou, Dian Cheng, Xuecheng Wang, Yizhang Wu, Baowei Zhang, Fenghua Fan, Shuo Wang, Bing Yang

**Affiliations:** Department of Cardiology, Shanghai East Hospital, Tongji University School of Medicine, Shanghai, China

**Keywords:** atrial fibrillation, left atrial appendage occlusion, radiofrequency ablation, one-stop procedure, direct current cardioversion

## Abstract

**Background and aims:**

Data on the safety of direct current cardioversion (DCCV) in patients with left atrial appendage occlusion (LAAO) devices and its impact on thromboembolic prevention are limited. This study aimed to investigate the safety and efficacy of DCCV in patients with LAAO devices.

**Methods:**

This single-center, ambispective cohort included patients undergoing one-stop procedures [LAAO combined with radiofrequency catheter ablation (RFCA)], where LAAO was performed first. DCCV was performed to restore sinus rhythm after LAAO. Patients were divided into the DCCV group and the no-DCCV group. Safety endpoints included DCCV-related death, device dislodgment, device embolization, and major bleeding events. Efficacy endpoints contained all-cause death, cardiovascular death, stroke/transient ischemic attack, and systemic embolism.

**Results:**

A total of 196 patients (age 72.5 ± 7.4 years, 51.0% male) were enrolled, with 95 patients undergoing DCCV after LAAO. No DCCV-related death, device dislodgement, or device embolism was observed. At 12 months, the safety endpoints occurred in 3.2% of the DCCV group vs. 6.9% of the no-DCCV group (*p* = 0.238). Similarly, the efficacy endpoints were observed in 1.1% of the DCCV group vs. 4.0% of the no-DCCV group (*p* = 0.339). By performing pre- and post-DCCV transesophageal echocardiography (TEE) in the prospective cohort, a significant increase in device diameter at 45° and 90° (*p* = 0.044; 0.027), and an insignificant decline trend of peri-device leak and shoulder at 135° were noted (*p* = 0.051; 0.103).

**Conclusions:**

No signal of excess risk was observed when performing DCCV in patients with LAAO devices. Tiny changes in device diameter after DCCV were noted on TEE at 45° and 90°, but these were not associated with adverse effects.

## Highlights

• **Competency in medical knowledge:** Performing direct current cardioversion (DCCV) in patients with LAAO devices is safe and does not reduce the efficacy of thrombosis prevention, even when done shortly after device implantation.

• **Competency in patient care:** In patients requiring DCCV, the presence of a recently implanted LAAO device should not be a reason to postpone cardioversion. Transesophageal echocardiography performed pre- and post-cardioversion can provide a detailed device change profile.

• **Translational outlook:** A multicenter, larger-scale prospective study would be helpful to confirm the safety and role of DCCV in patients with LAAO devices.

## Introduction

Atrial fibrillation (AF) is the most common sustained cardiac arrhythmia in adults worldwide and is associated with a four- to fivefold increased risk of ischemic stroke (1). Although oral anticoagulation (OAC) is recommended for thromboembolism prevention, bleeding complications and non-adherence are hard to ignore. Left atrial appendage occlusion (LAAO) has emerged as an alternative for the prevention of embolization in AF patients who are not candidates for long-term OAC (2).

Radiofrequency catheter ablation (RFCA) is an effective rhythm control strategy for patients with AF and has been recommended as first-line therapy (2). Direct current cardioversion (DCCV) is also an integral part of the management of AF in symptomatic patients requiring rhythm control (3). An increasing number of patients undergoing rhythm control are also being treated with LAAO (4, 5). Consequently, DCCV to restore sinus rhythm in patients with LAAO has become increasingly common (6, 7). However, in patients who have undergone LAAO, data on the safety of DCCV and its impact on the thrombosis prevention efficacy of LAAO remain limited.

## Method

### Study design and population

**Figure 1:**
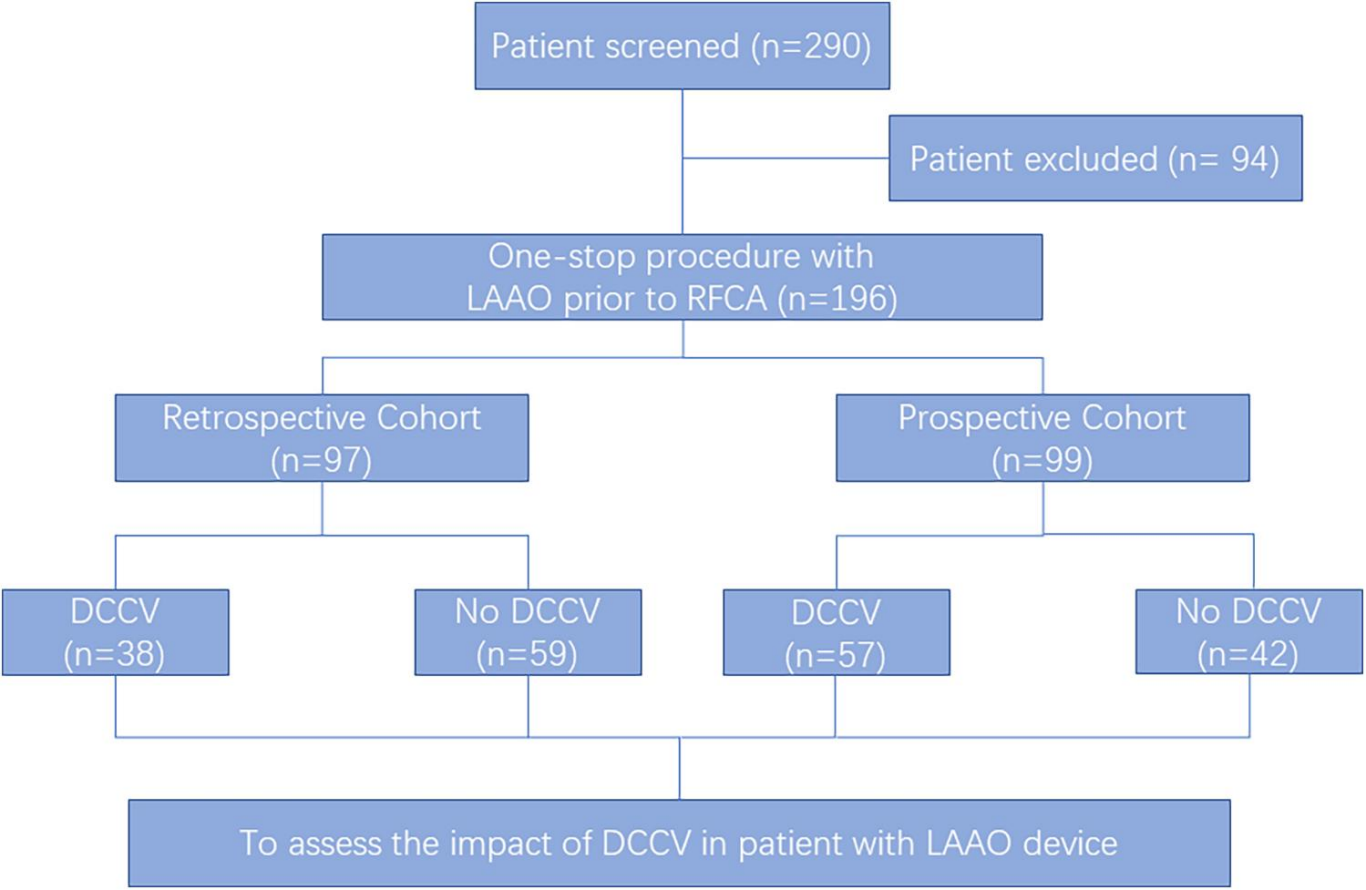
Participant selection. Of the 290 patients who underwent the one-stop procedure (left atrial appendage occlusion and radiofrequency ablation) in Shanghai East Hospital from February 2019 to February 2022, 196 patients were enrolled. LAAO, left atrial appendage occlusion; RFCA, radiofrequency catheter ablation; DCCV, direct current cardioversion.

This study was a single-center, ambispective cohort study conducted at Shanghai East Hospital from February 2019 to February 2022. It included patients who underwent one-stop procedures (LAAO combined with RFCA), in which LAAO was performed prior to RFCA. Eligible patients were aged ≥18 years and scheduled to undergo the one-stop procedure. The exclusion criteria included receiving RFCA before LAAO during the same procedure, procedures performed under fluoroscopic guidance only, and implantation of any non-Watchman (WM) device (Watchman, Boston Scientific, Marlborough, MA, USA). The study was approved by the institutional ethics committee of Shanghai East Hospital and complied with the Declaration of Helsinki. The study aimed to investigate the safety of DCCV after the LAAO procedure and its impact on the thrombosis prevention efficacy of LAAO. Procedures performed before 20 November 2020 constituted the retrospective cohort, and those performed after constituted the prospective cohort. The sequence of the one-stop procedure was determined by a physician. In the retrospective cohort, the position and morphology of the LAAO device were confirmed by intraprocedural fluoroscopy pre- and post-DCCV. In the prospective cohort, LAAO device parameters were measured under transesophageal echocardiography (TEE) pre- and post-DCCV. Patient enrollment is shown in Figure 1. Baseline information, procedure patterns, device parameters, and long-term outcomes were systematically collected.

### One-stop procedure

All patients were anticoagulated, and left atrial thrombus was excluded by TEE or cardiac computed tomography angiography (CCTA) before operation. All procedures were performed under general anesthesia, and the procedural sequence was determined by the operator. Intravenous heparin was administered to maintain an activated clotting time of 300 ± 50 s.

LAAO procedures were performed under fluoroscopy and TEE guidance. Amiodarone was routinely administered intravenously in non-paroxysmal atrial fibrillation (NPAF) patients. Vascular access was obtained via bilateral femoral veins. An 8.5 F sheath (SL1, St. Jude Medical, MN, USA) was used for the transseptal puncture, which was then replaced by the LAAO delivery system. LAA anatomy parameters, including morphology, orifice diameter, and depth, were measured by fluoroscopy and TEE, and the size of the WM device was correspondingly selected. The device was delivered and unsheathed into the LAA via delivery system, and the “PASS” criteria were all met before release (8). Angiographic and TEE assessments were subsequently performed.

After LAAO, a decapolar catheter was advanced into the coronary sinus, and a quadripolar catheter was placed at the His bundle region. For patients who remained in AF, DCCV was performed using synchronous biphasic current (200 J). Patch placement was standardized to the anterolateral position. In the retrospective cohort, the pre- and post-DCCV device position and morphology were confirmed by fluoroscopy, while in the prospective cohort, pre- and post-DCCV measurements were performed by TEE. If the patient remained AF after one DCCV, performing sequential DCCVs or RFCA would depend on the physician's decision.

Mapping and ablation were performed under the guidance of the CARTO system (Biosense Webster, CA, USA). As previously described (9), pulmonary vein isolation (PVI) was performed first, and corresponding additional ablations were performed according to the intraprocedural arrhythmia episode, provocation, and mapping result.

### Definitions

DCCV-related safety endpoints were defined as a composite of DCCV-related death, device dislodgment, device embolization, and major bleeding events [bleeding score >3 points, as defined by the Bleeding Academy (BARC)] (10). Thrombosis prevention efficacy endpoints included all-cause death, cardiovascular death, stroke/transient ischemic attack (TIA), and systemic embolism. Successful DCCV was defined as the presence of sinus rhythm on a 12-lead ECG recorded 1 min after cardioversion (11). Clinical composite endpoints of major adverse cardiovascular and cerebrovascular events (MACCE) included all-cause death, heart failure (HF)-related rehospitalization, stroke/TIA, systemic embolism, acute coronary syndrome (ACS), and major bleeding events. Device-related thrombosis (DRT) was defined as a homogenous echo-dense mass visible in multiple planes with independent motion and adherence to the atrial surface of the LAAO device on TEE or a significant hypoattenuated thickening on the atrial surface of the LAAO device (12). Complete device endothelialization (CDE) was defined as LAA attenuation <100 HU or LAA/left atrium attenuation ratio ≤0.25 and no trans-fabric leak on cardiac CT at 3 months post-procedure (13).

### Follow-up

OAC therapy and antiarrhythmic drugs were prescribed for all patients for at least 3 months after the procedure. Patients were followed up through clinic visits at 1, 3 (the blanking period), 6, and 12 months. TEE or CCTA was performed at the 3-month follow-up to assess the device position, peri-device leak (PDL), thrombus formation, and CDE. In patients without PDL ≥5 mm, OAC was discontinued, and dual antiplatelet therapy with aspirin and clopidogrel was prescribed till the 6th month post-procedure. Thereafter, single antiplatelet therapy was continued indefinitely unless contraindicated (14). Holter recording and echocardiography were conducted at every follow-up visit. Pulse measurement and electrocardiogram (ECG) recording were recommended whenever patients were symptomatic. Successful ablation was defined as no atrial tachyarrhythmia ≥30 s after the blanking period off antiarrhythmic drugs (15).

### Statistical analysis

The continuous variables were expressed as mean ± SD, while categorical variables were expressed as number and percentage. Continuous variables were compared using an unpaired *t*-test or Wilcoxon rank-sum test, while categorical variables were compared using the chi-square test or Fisher's exact test. The event-free rates were calculated using Kaplan–Meier analysis, while log-rank statistics were used for group comparisons. Univariate and multivariable logistic regression analyses were performed to assess independent predictors associated with CDE, and Cox regression analysis was performed to determine the predictors of AF recurrence. Multivariable logistic regression model and propensity score (PS) analyses were performed to improve comparability between groups. The results are expressed as *p*-values. Factors with *p* < 0.1 in univariate analyses were enrolled in multivariate analyses. A *p*-value of <0.05 was considered statistically significant. All statistical analyses were performed using SPSS software version 20.0.

## Results

### Baseline characteristics

A total of 290 patients underwent the one-stop procedure from February 2019 to February 2022, of whom 94 were excluded based on the study’s exclusion criteria. As a result, 196 patients were enrolled in this study (Figure 1), of whom 97 patients were retrospectively enrolled, and 99 were prospectively enrolled. Of the patients who underwent LAAO first, 95 patients underwent DCCV after LAAO (DCCV group, G_D_), and 101 were not (no-DCCV group, G_ND_). The mean age was 72.5 ± 7.4 years, and 51.0% were male. NPAF accounted for 55.1% of all patients, and 88.3% underwent an index procedure. The mean CHA_2_DS_2_-VASc and HAS-BLED scores were 4.3 ± 1.6 and 2.2 ± 1.0, respectively (Table 1).

**Table 1:** Baseline characteristics.

Baseline characteristics	All patients (*N* = 196)	G_D_ (*n* = 95)	G_ND_ (*n* = 101)	*p*
Age, years	72.5 ± 7.4	71.7 ± 7.2	7.3.3 ± 7.5	0.14
Male	100 (51.0)	49 (51.6)	51 (50.5)	0.88
Course of AF, month	51.8 ± 63.9	52.2 ± 66.1	51.3 ± 62.1	0.93
NPAF	108 (55.1)	77 (81.1)	31 (30.7)	<0.01
Smoke	35 (17.6)	16 (16.8)	19 (18.8)	0.72
Alcohol consumption	22 (11.2)	13 (13.7)	9 (8.9)	0.29
Hypertension	138 (70.4)	70 (73.7)	68 (67.3)	0.33
Diabetes	68 (34.7)	30 (31.6)	38 (37.6)	0.37
CAD	57 (29.1)	27 (28.4)	30 (29.7)	0.84
Myocardial infarction	6 (3.1)	5 (5.3)	1 (1.0)	0.11
Chronic heart failure	60 (30.6)	28 (29.5)	32 (31.7)	0.74
Index procedure	173 (88.3)	90 (92.6)	83 (84.2)	0.07
CHA_2_DS–-VASc score	4.3 ± 1.6	4.3 ± 1.6	4.4 ± 1.6	0.66
HAS-BLED score	2.2 ± 1.0	2.3 ± 1.0	2.2 ± 1.0	0.32
LAD, mm	43.3 ± 5.1	45.3 ± 5.0	41.4 ± 4.5	<0.01
LVDd, mm	47.3 ± 4.7	48.0 ± 4.9	46.6 ± 4.6	0.05
LVDs, mm	30.8 ± 4.8	31.7 ± 5.0	29.9 ± 4.4	<0.01
LVEF, %	63.5 ± 7.4	62.4 ± 7.0	64.6 ± 7.7	0.04

Values are mean ± SD or *n* (%). AF, atrial fibrillation; NPAF, non-paroxysmal atrial fibrillation; CAD, coronary artery disease; CHA_2_DS_2_-VASc, congestive heart failure, hypertension, age ≥75 years, diabetes mellitus, prior stroke or transient ischemic attack or thromboembolism, vascular disease, age 65–74 years, sex category; HAS-BLED, hypertension, abnormal renal and liver function, stroke, bleeding tendency or predisposition, labile INRs, elderly, drugs; LAD, left atrium diameter; LVDd, left ventricular end-diastolic dimension; LVDs, left ventricular end-systolic diameter; LVEF, left ventricular ejection fraction; G_D_, DCCV group; G_ND_, no-DCCV group; DCCV, direct current cardioversion.

Compared with G_ND_, G_D_ had a higher prevalence of NPAF (81.1% vs. 30.7%, *p* < 0.01), larger left atrium diameter (45.3 ± 5.0 mm vs. 41.4 ± 4.5 mm, *p* < 0.01), larger left ventricular end-systolic dimension (31.7 ± 5.0 mm vs. 29.9 ± 4.4 mm, *p* < 0.01), and lower left ventricular ejection fractions (LVEF, 62.4 ± 7.0% vs. 64.6 ± 7.7%, *p* = 0.04). A higher incidence of index procedure (92.6% vs. 84.2%, *p* = 0.07) was also observed in G_D_. Detailed comparisons between the two groups are summarized in Table 1.

### Procedural characteristics

The average procedure time and fluoroscopy time were 246.6 ± 65.8 and 14.9 ± 6.0 min, respectively. Before device implantation, all LAA were assessed by TEE. Cauliflower, windsock, cactus, and chicken wing account for 49 (25.0%), 54 (27.6%), 63 (32.1%), and 30 (15.3%) of all patients. Complete occlusion of LAA was confirmed by TEE in 148 (75.5%) patients, while PDL of <3 and 3–5 mm was observed in 44 (22.4%) and 4 (2.0%) patients. No PDL of ≥5 mm was detected after release. The 27 mm device was the most used size in the study, accounting for 52 (26.5%) patients. Compared with G_ND_, G_D_ had a larger maximum and minimum LAA ostium width (23.7 ± 3.4 mm vs. 22.5 ± 3.9 mm, *p* = 0.03; 19.9 ± 3.2 mm vs. 18.7 ± 3.6 mm, *p* = 0.02) and a lower minimum device compression (16.6 ± 5.2% vs. 18.3 ± 5.8%, *p* = 0.03). No significant difference was noted in procedure and fluoroscopy time, LAA morphology, PDL, device size, and complications between the two groups (Table 2).

**Table 2:** Procedural characteristics.

Procedural characteristics	All patients (*N* = 196)	G_D_ (*n* = 95)	G_ND_ (*n* = 101)	*p*
Procedure time, min	246.6 ± 65.8	241.6 ± 53.6	251.7 ± 76.2	0.30
Fluoroscopy time, min	14.9 ± 6.0	15.3 ± 6.5	14.5 ± 5.5	0.47
Morphology of LAA				
Cauliflower	49 (25.0)	20 (21.1)	29 (28.7)	0.61
Windsock	54 (27.6)	28 (29.5)	26 (25.7)	
Cactus	63 (32.1)	33 (34.7)	30 (29.7)	
Chicken wing	30 (15.3)	14 (14.7)	16 (15.8)	
Max LAA ostium width, mm	23.1 ± 3.7	23.7 ± 3.4	22.5 ± 3.9	0.03
Min LAA ostium width, mm	19.3 ± 3.4	19.9 ± 3.2	18.7 ± 3.6	0.02
Max device compression, %	23.3 ± 5.6	22.7 ± 5.7	23.9 ± 5.6	0.15
Min device compression, %	17.5 ± 5.6	16.6 ± 5.2	18.3 ± 5.8	0.03
Peri-device leak at implantation				
Complete occlusion	148 (75.5)	76 (80.0)	72 (71.3)	0.33
Leak <3 mm	44 (22.4)	17 (17.9)	27 (26.7)	
Leak 3–5 mm	4 (2.0)	2 (2.1)	2 (2.0)	
Leak >5 mm	0 (0.0)	0 (0.0)	0 (0.0)	
Size of the WM device				
21 mm	9 (4.6)	3 (3.2)	6 (5.9)	0.62
24 mm	44 (22.4)	18 (18.9)	26 (25.7)	
27 mm	52 (26.5)	26 (27.4)	26 (25.7)	
30 mm	50 (25.5)	27 (28.4)	23 (22.8)	
33 mm	41 (20.9)	21 (22.1)	20 (19.8)	
Complications				
Pericardial effusion	1 (0.5)	0 (0.0)	1 (1.0)	1.00
Stroke/TIA	1 (0.5)	0 (0.0)	1 (1.0)	1.00
Bleeding	5 (2.6)	1 (1.1)	4 (4.0)	0.37
Complications of vascular access	6(3.1)	4(4.2)	2(2.0)	0.43

Values are mean ± SD or *n* (%). LAA, left atrial appendage; TIA, transient ischemic attack; G_D_, DCCV group; G_ND_, no-DCCV group; DCCV, direct current cardioversion.

### DCCV characteristics

A total of 102 DCCVs were performed in 95 patients, following the LAAO procedure. Eight (8.4%) patients failed to restore sinus rhythm or converted to AF within 1 min after DCCV; all were successfully converted to sinus rhythm after RFCA. Among the 95 patients, 4 (4.2%) presented sinus arrest after DCCV, and a temporary cardiac pacemaker was used in 2 patients. All patients restored to sinus rhythm at discharge, and no permanent pacemaker was implanted.

### Clinical outcome

**Figure 2:**
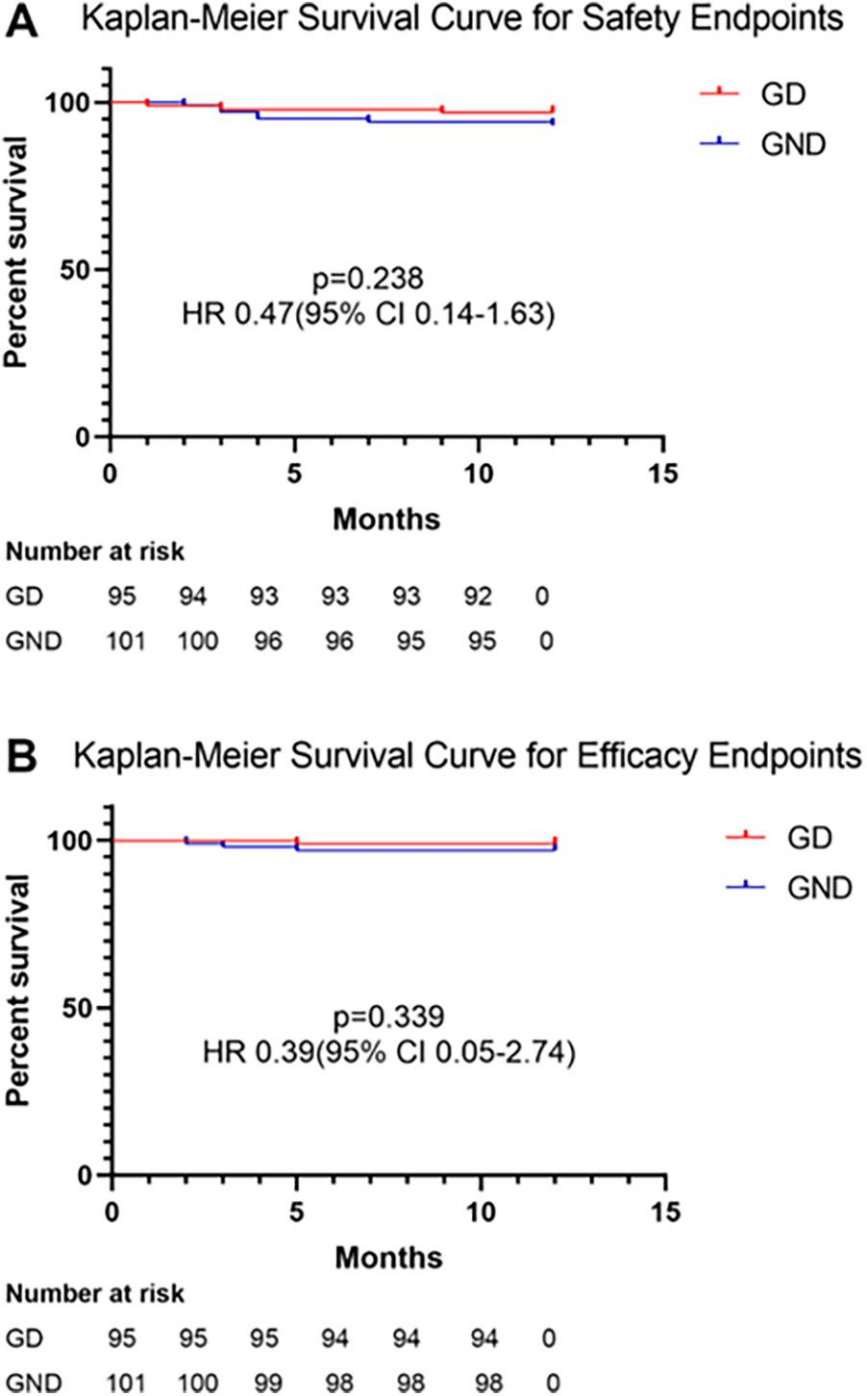
Kaplan–Meier survival curve for **(A)** safety and **(B)** efficacy Endpoints.

Regarding safety endpoints, no DCCV-related death, device dislodgment, or device embolization occurred during the perioperative period. Device dislodgment was noted in one patient (0.5%) during follow-up in the retrospective group; this patient received no DCCV in the procedure. Major bleeding happened in nine (4.6%) patients, of whom eight had gastrointestinal bleeding and one had urinary hemorrhage. No fatal bleeding happened during follow-up. No significant difference was noted between G_D_ and G_ND_ (3.2% vs. 6.9%, *p* = 0.238, Figure 2). LAAO efficacy endpoints were assessed during follow-up. One patient (0.5%) died due to refractory heart failure. Stroke/TIA occurred in three patients (1.1%), and no systemic embolism was noted. There was no significant difference between the two groups (1.1% vs. 4.0%, *p* = 0.339, Figure 2). After multivariable adjustment, inverse probability of treatment weighting (IPTW), and PS matching, DCCV was not significantly associated with adverse safety or efficacy outcomes (Supplementary Table S1).

Twenty (10.2%) MACCE occurred, including five HF, two ACS, one all-cause death, three stroke/TIA, and nine major bleeding events. The MACCE between G_D_ and G_ND_ was comparable (6 vs. 14, *p* = 0.08). At the 1-year follow-up, AF recurred in 30 (15.3%) patients. Due to the larger portion of the NPAF population, G_D_ presented a significantly higher AF recurrence (22.1% vs. 8.9%, *p* = 0.01).

### LAAO characteristics

TEE/CCTA data were acquired from 156 patients, of whom 120 (77%) underwent CCTA reassessment, 22 (14%) underwent TEE recheck, and 14 (9%) received both. DRT was found in two (1.0%) patients, one of whom had undergone DCCV. Complete occlusion of LAA, <3 mm PDL, and 3–5 mm PDL were noted in 92 (59.0%), 50 (32.1%), and 13 (8.3%) patients, respectively. No significant difference was noted between G_D_ and G_ND_ (Table 3). Owing to the dislodgment of the device, one patient presented a PDL over 5 mm, while device embolization did not occur. Discontinued OAC therapy was prescribed, and no stroke/TIA or systemic embolism was observed.

**Table 3:** LAAO follow-up characteristics.

Imaging assessment	All patients (*N* = 196)	G_D_ (*n* = 95)	G_ND_ (*n* = 101)	*p*
TEE/CCTA data available	156	75	81	0.83
DRT	2 (1.3) (*N* = 156)	1 (1.3) (*n* = 75)	1 (1.2) (*n* = 81)	1.00
Complete occlusion of the LAA	92 (59.0) (*N* = 156)	39 (52.0) (*n* = 75)	53 (65.4) (*n* = 81)	0.17
Residual flow (<3 mm)	50 (32.1) (*N* = 156)	30 (40.0) (*n* = 75)	20 (24.7) (*n* = 81)	
Residual flow (3–5 mm)	13 (8.3) (*N* = 156)	6 (8.0) (*n* = 75)	7 (8.6) (*n* = 81)	
Residual flow (>5 mm)	1 (0.6) (*N* = 156)	0 (0.0) (*n* = 75)	1 (1.2) (*n* = 81)	
CDE	46 (34.3) (*N* = 134)	19 (30.2) (*n* = 63)	27 (38.0) (*n* = 71)	0.34

Values are *n* (%). TEE, transesophageal echocardiography; CCTA, cardiac computed tomography angiography; DRT, device-related thrombus; LAA, left atrial appendage; CDE, complete device endothelialization; G_D_, DCCV group; G_ND_, no-DCCV group; DCCV, direct current cardioversion.

CDE was assessed in a patient who underwent a CCTA exam. Among 134 patients with CCTA images, 46 (34.3%) reached CDE, presenting LAA attenuation <100 HU or LAA/left atrium attenuation ratio ≤0.25 and no trans-fabric leak on CCTA. In G_D_, 19 (30.2%) patients achieved CDE and presented no significant difference with those in G_ND_ (30.2% vs. 28.0%, *p* = 0.34).

In the prospective cohort, pre- and post-TEE was performed to quantitatively analyze the impact of DCCV on the LAAO device. After DCCV, device diameter significantly increased at TEE 45° and 90° (45° pre vs. post, 22.4 ± 2.9 mm vs. 22.8 ± 2.8 mm, *p* = 0.04; 90° pre vs. post, 22.6 ± 3.1 mm vs. 23.0 ± 2.9 mm, *p* = 0.03). Accordingly, device compression rate at 90° significantly decreased (90° pre vs. post, 19.8 ± 6.3% vs. 18.3 ± 5.7%, *p* = 0.02), and a non-significant declining trend was observed at 45° (45° pre vs. post, 20.4 ± 6.4% vs. 19.2 ± 5.7%, *p* = 0.05). Meanwhile, after DCCV, the device PDL and shoulder at 135° showed a declining trend as well (135° PDL pre vs. post, 0.5 ± 1.0 mm vs. 0.3 ± 0.8 mm, *p* = 0.05; 135° shoulder pre vs. post, 3.5 ± 3.3 mm vs. 3.0 ± 2.8 mm, *p* = 0.10). Detailed parameters are presented in Table 4.

**Table 4:** TEE evaluation on LAAO device pre- and post-DCCV.

TEE assessment	Before DCCV	After DCCV	*p*
Device diameter, mm			
0°	22.6 ± 2.9	22.8 ± 3.0	0.31
45°	22.4 ± 2.9	22.8 ± 2.8	0.04
90°	22.6 ± 3.1	23.0 ± 2.9	0.03
135°	23.2 ± 3.2	23.2 ± 2.9	1.00
Device compression, %			
0°	19.7 ± 6.2	19.0 ± 6.0	0.32
45°	20.4 ± 6.4	19.2 ± 5.7	0.05
90°	19.8 ± 6.3	18.3 ± 5.7	0.02
135°	17.7 ± 5.9	17.7 ± 5.9	0.89
Peri-device leak, mm			
0°	0	0	/
45°	0	0	/
90°	0.1 ± 0.5	0.1 ± 0.3	0.37
135°	0.5 ± 1.0	0.3 ± 0.8	0.05
Device shoulder, mm			
0°	0.5 ± 1.8	0.8 ± 1.9	0.19
45°	0.8 ± 1.8	0.8 ± 1.8	0.99
90°	1.5 ± 2.8	1.1 ± 2.4	0.14
135°	3.5 ± 3.3	3.0 ± 2.8	0.10

Values are mean ± SD.

DCCV, direct current cardioversion; TEE, transesophageal echocardiography.

## Discussion

The current ambispective cohort study demonstrates the safety and feasibility of DCCV performed shortly after the LAAO procedure, and may provide valuable information to the current existing evidence gap. Firstly, DCCV after LAAO is a safe operation in clinical practice. No DCCV-related death, device dislodgment, or embolism was observed. During follow-up, the thromboembolism prevention efficacy of LAAO and the incidence of MACCE were not affected by DCCV. Secondly, the DCCV's impact on device position and morphology was evaluated by TEE pre- and post-DCCV. We noted that the device diameter at 45° and 90° increased after DCCV, while PDL and shoulder at 135° showed a trend of improvement. Finally, DCCV performed shortly after the LAAO procedure was not associated with a significant influence on PDL and CDE in the follow-up (Central Illustration).

**Central Illustration:**
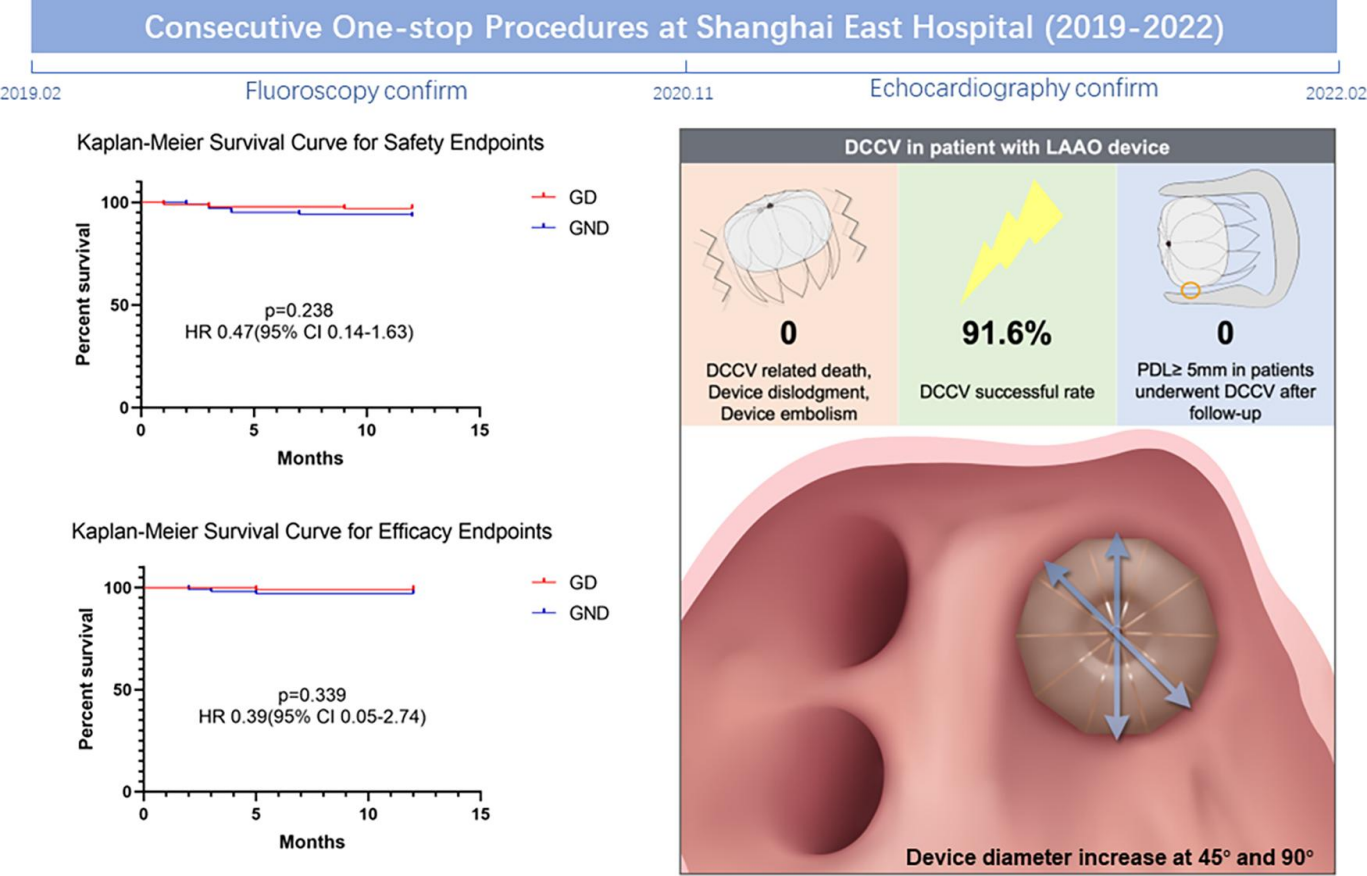
Comprehensive images presenting the impact of DCCV on patients with the LAAO device. DCCV, direct current cardioversion; LAAO, left atrial appendage occlusion.

LAAO is gaining ground as an alternative choice for anticoagulation, especially in patients with contraindications for OAC (2). DCCV is a common procedure for terminating AF and plays an important role in the rhythm control (1, 16). However, both guideline recommendations and safety data on DCCV in patients with LAAO remain limited (3). Hanazawa et al. (17) first reported a case of stroke after DCCV in a patient with an LAAO device (2 years post-implantation), who had evidence of narrow residual flow and no intracardiac thrombi detected by TEE before DCCV. Phillips et al. (18) later described a single-center experience in 13 patients who underwent DCCVs in a cohort of 98 patients treated with combined RFCA and LAAO, ranging from 9 days to 18 months after the index procedure, with no detectable dislodgement observed. Subsequently, Berte et al. (7) further reported that 41 DCCVs were performed in 26 patients after a mean follow-up of 17 ± 17months, and no stroke or TIA occurred. Sharma et al. (6) reported the largest population of patients who underwent DCCV with the LAAO device. A total of 148 patients were retrospectively collected from a multicenter study. The median duration of DCCV post-LAAO was 5.1 months, and none of the patients had a PDL of ≥5 mm, incomplete device apposition, or embolization after DCCV. Maarse et al. (19) published their prospective multicenter registry study involving 93 patients who underwent 284 DCCVs, ranging from 0 days to 8 years post-LAAO. Two device rotations and device embolization were reported, but their association with DCCV was uncertain due to the lack of an image before the operation. These data suggested that performing DCCV with the LAAO device is a relatively safe operation. However, several deficiencies should be noted. For example, the duration between DCCV and LAAO implantation is highly variable, ranging from days to years, leaving the impact of endothelialization and other time-dependent factors ignored. Moreover, the lack of pre- and post-image made the association between adverse events and DCCV operations unclear, leaving detailed device position change unexplored. In our study, DCCV was performed shortly after the LAAO procedure. Thus, the endothelialization and other potential influencing factors could be evaded, making the observation of operation safety more direct. As described, this procedure, performed shortly after DCCV, is safe and shows no adverse effect on thrombosis prevention efficacy. Accordingly, after 45–90 days of endothelialization (20), the device would be more firmly attached to the LAA, making the later performed DCCV further safer. Therefore, being safe is an inherent feature of DCCV in patients with LAAO devices. The time gap between device implantation and DCCV should not be the reason to postpone such an operation, especially in those patients with unstable hemodynamics.

The position and morphology change after DCCV were not evaluated in a previous study (6, 7, 18, 19). Although seldom device dislodgment or embolism were noted after DCCV, the tiny change still could happen. By performing TEE pre- and post-DCCV, we find that the diameter at 45° and 90° significantly increased, and the PDL and shoulder at 135° showed a decreasing trend. The extent of this change is small, and no obvious morphology change was noted by fluoroscopy. Silva et al. (21) reported two cases presenting position and morphological change after release, and named this phenomenon the “popcorn effect.” Compared with the change induced by DCCV, the “popcorn effect” presents several different points. Firstly, the “popcorn effect” has a greater extent of position and morphology change, which could be observed by fluoroscopy. While the DCCV-induced change is tiny, and almost cannot be noted by regular fluoroscopy. Secondly, “popcorn effect” represents a high-tension device restored in LAA after release and may be linked to device embolism. The change induced by DCCV, in our study, was not observed to be associated with adverse events. On the contrary, performing TEE showed a trend to improve PDL and shoulder at 135°. This tiny change acts more like a self-adaptation with the anatomy of LAA, rather than a compression pressure release.

In the follow-up, the incidence and extent of PDL in patients who underwent DCCV did not significantly differ from those free of DCCV. Over 40% of patients are still presenting mild to moderate PDLs. Although PDL ≤5 mm is the criterion of switching OAC to dual antiplatelet therapy, recent long-term analyses have demonstrated that peri-device leaks as small as 3–5 mm are independently associated with increased thromboembolic events after LAAO. In our cohort, persistent leaks of this magnitude were observed in 8.3% of patients at 3–6 months. Although the absolute event rate remained low and was similar between the DCCV and no-DCCV groups, this underscores the importance of meticulous device sizing and implantation technique. Future studies with larger populations and longer follow-up will be necessary to determine whether early DCCV influences the evolution or clinical impact of these small leaks (22). CDE was evaluated by CCTA in our study; 34.3% of patients reached CDE at 6-month follow-up, which was similar to previous reports (23–25). While studies have shown that incomplete device endothelialization increases the risk of thromboembolic events and DRT (24). Similar to its impact on PDL, DCCV seems to have no adverse effect on CDE. Although presenting an adaptive position change effect, DCCV could not improve the progress of CDE either.

## Limitations

First, regarding study design, this is an ambispective cohort study, which is essentially an observational study and includes a retrospective cohort, so there may be bias and influence of unmeasured confounding factors on the result. The prospective cohort may make up for this deficiency to a certain extent. Secondly, a relatively small sample size and non-randomized design made the study underpowered. Although multivariable regression, IPTW, and PS matching were applied, residual confounding may still exist due to the non-randomized design. However, similar to other studies, a randomized design would be especially challenging due to the low incidence of adverse events (6). Thirdly, our study exclusively included patients implanted with the Watchman device. Therefore, the findings may not be generalizable to other LAAO devices that differ in frame design, anchoring mechanism, or endothelialization characteristics. Finally, CCTA is preferred in reexamination during follow-up, which may result in a discrepancy with parameters assessed by TEE. To reduce this bias, CDE was only assessed in patients with CCTA data, and a small proportion of TEE applies in PDL measurement may reduce bias as well.

## Conclusions

No signal of excess risk was observed when performing DCCV in patients with an LAAO device, although wide confidence intervals and limited events preclude definitive conclusions. The time gap between the LAAO procedure and DCCV should not be regarded as a reason to postpone rhythm control. While DCCV may cause a tiny change in the device at 45° and 90° on TEE, no associated complication was noted in the current study. Larger, randomized controlled trials are warranted to further validate the safety of DCCV in these patients.

## Data Availability

The raw data supporting the conclusions of this article will be made available by the authors, without undue reservation.
