# Supplementary material for: Direct current cardioversion of atrial fibrillation in patients with left atrial appendage occlusion devices
Source: Front Cardiovasc Med. 2025 Nov 5;12:1604268. doi: 10.3389/fcvm.2025.1604268 (PMC12626986; doi:10.3389/fcvm.2025.1604268)
Supplement: Supplementary file 1 [file Table1.docx]

| Analysis Method | Safety OR (95% CI) | p-value | Efficacy OR (95% CI) | p-value |
| --- | --- | --- | --- | --- |
| Multivariable Logistic | 0.84 (0.15–4.70) | 0.84 | 0.48 (0.03–7.99) | 0.61 |
| IPTW Logistic | 0.63 (0.22–1.76) | 0.38 | 0.25 (0.03–2.23) | 0.22 |
| PS Matching | 0.35 (0.09–1.38) | 0.13 | – (too few events) | – |

Supplementary Table S1 Impact of DCCV on Safety and Efficacy Endpoints (Multivariate and Propensity Score Analysis)
